# Antibodies against outer-capsid proteins of grass carp reovirus expressed in *E. coli *are capable of neutralizing viral infectivity

**DOI:** 10.1186/1743-422X-8-347

**Published:** 2011-07-12

**Authors:** Ling Shao, Xiaoyun Sun, Qin Fang

**Affiliations:** 1 State Key Laboratory of Virology, Wuhan Institute of Virology, Chinese Academy of Sciences, Wuhan 430071, China; 2 Graduate School of the Chinese Academy of Sciences, Beijing 100039, China

## Abstract

**Background:**

Grass carp reovirus (GCRV), which causes severe infectious outbreaks of hemorrhagic disease in aquatic animals, is a highly pathogenic agent in the *Aquareovirus *genus of family *Reoviridae*. The outer capsid shell of GCRV, composed of the VP5-VP7 protein complex, is believed to be involved in cell entry. The objective of this study was to produce a major neutralization antibody for mitigating GCRV infection.

**Results:**

Recombinant plasmids of GCRV outer capsid proteins VP5 and VP7 were constructed and expressed in prokaryotic cells in our previous work. In this study, we prepared GCRV Antibody (Ab), VP5Ab and VP7Ab generated from purified native GCRV, recombinant VP5 and VP7 respectively. Immunoblotting analysis showed that the prepared antibodies were specific to its antigens. In addition, combined plaque and cytopathic effect (CPE)-based TCID_50 _(50% tissue culture infective dose) assays showed that both VP5Ab and VP7Ab were capable of neutralizing viral infectivity. Particularly, the neutralizing activity of VP7Ab was 3 times higher than that of VP5Ab, suggesting that VP7 might be a dominating epitope. Moreover, the combination of VP5Ab and VP7Ab appeared to enhance GCRV neutralizing capacity.

**Conclusions:**

The results presented in this study indicated that VP7 protein was the major epitope of GCRV. Furthermore, VP5Ab and VP7Ab in combination presented an enhanced capacity to neutralize the GCRV particle, suggesting that the VP5 and VP7 proteins may cooperate with each other during virus cell entry. The data can be used not only to further define the surface epitope domain of GCRV but may also be applicable in the designing of vaccines.

## Background

Grass carp reovirus (GCRV), a member of genus *Aquareovirus *in the family *Reoviridae*[1], was the first viral pathogen to be identified from aquatic animals in China; this virus was identified twenty years ago during an acute epidemic characterized by symptoms of hemorrhagic disease in fingerling and yearling grass carp [2,3]. In an attempt to control the spread of this disease, progress has been made using a crude inactive vaccine preparation, but the agent is far from being effective in the prevention of GCRV viral infection. In addition, GCRV has been recognized as the most pathogenic of the isolated aquareoviruses reported to date [4,5]. Therefore, it is important to develop an effective vaccine for better prevention and control of fatal outbreaks of hemorrhagic disease.

The GCRV is a nonenveloped icosahedral particle comprising 11 double-stranded RNA genome segments surrounded by multiple concentric protein capsids [6]. The 11 genome segments encode seven structural proteins (VP1-VP7) and five nonstructural proteins [7,8]. Although the 11-part segmented genome of GCRV is similar in composition to members of the genus *Rotavirus *within the family *Reoviridae*, there is no genetic relationship with rotavirus based on reciprocal RNA-RNA dot blot hybridization [9,10]. Previous biological studies have indicated that the GCRV can produce a typical cytopathic effect (CPE) with large syncytia in its sensitive cells [11]. In addition, the virions are resistant to chloroform and ether, insensitive to acid (pH 3) and alkaline (pH 10) treatment; they are also stable within an extensive range of temperatures [12], which suggest that the virus is very stable in harsh natural environments. Recent cryo-electron microscopy (cryo-EM) and three-dimensional (3D) structural reconstruction images indicate that the inner layer arranges with a *T *= 1 symmetry. This layer is composed of 5 proteins (including VP1-VP4 and VP6) and possesses the enzymatic activities necessary for viral transcription [6,7,13,14]. The other outer capsid proteins, arranged on an incomplete *T *= 13 icosahedral lattice, are composed of VP5 and VP7; each GCRV virion contains 200 trimers formed by VP5-VP7 heterodimers, a structure homologous to the μ1_3_σ3_3 _complex of MRV (Mammalian reovirus)[13,15]. Similar to μ1 protein in MRV, the VP5 protein can exist in two conformations in virions and infectious subviral particle (ISVP); these conformations are thought to be caused by autocleavage near the N-terminus between amino acid residues Asn42 and Pro43 [16-20]. Protein VP7, the major surface protein of virions, adopts icosahedral positions through its close interactions with underlying VP5 subunits, providing stability for the virion or VP5 protein, a function similar to that of σ3 protein of MRV [13]. However, GCRV lacks a counterpart to the MRV protein σ1, which functions as a cell attachment protein and is situated on each fivefold vertex [7,13]. Meanwhile, biological experiments have shown that the complete digestion of VP7 and partial cleavage of VP5 lead to enhanced infectivity, suggesting that VP7 and VP5 may play important roles in virus entry into cells [16]. Notably, recent progress on the GCRV infectious subviral particle 3.3 Å atom structure revealed a priming mechanism of dsRNA virus entry into cells [17].

To gain insight into defining the GCRV major neutralization epitope and find an effective way to inhibit viral infection, the effects of neutralizing antibodies directed against VP5 and VP7 proteins were investigated in this study. Previously, we constructed the recombinant outer capsid proteins VP5 and VP7 of GCRV and expressed His-tag fusion proteins in *E. coli *[21,22]. In the present work, we expressed His-tag fusion VP5 and VP7 protein in a large scale and then purified the fusion proteins. In addition, we examined the neutralization ability of the GCRV antibody, VP5Ab, VP7Ab individually, and VP5Ab and VP7Ab in combination, respectively. We found that the specific GCRV antibody, VP5Ab and VP7Ab are capable of neutralizing viral infectivity. Particularly, the neutralizing activity of VP7 antiserum is 3 times higher than that of VP5, suggesting the VP7 might be the dominating epitope. Moreover, the combination of VP5 and VP7 antibodies showed enhanced neutralizing capacity to GCRV. The result reported in this study can help not only to further define GCRV surface epitopes, but they may also contribute toward the design of subunit vaccines.

## Methods

### Cell and virus

The CIK(*Ctenopharyngodon idellus *kidney)cell line, established by Zuo *et. al *[23], was used for the propagation of grass carp reovirus (GCRV). The GCRV873 strain, maintained in the author's laboratory, was used in this study [11,12]. The cells and viruses were cultivated according to previously described methods [11].

### Virus purification and transmission electron microscopy

GCRV particles from infected culture supernatant were purified using an established method [6]. The concentrations of purified viral particles from virus infected cell supernatants were measured using a spectrophotometer (Biotek, USA) following the instruction manual of the instrument. For examination by transmission electron microscopy (TEM), the purified viral particles were negatively stained with 3% (w/v) phosphotungstic acid (PTA, pH 6.8). All the sample grids were examined by transmission electron microscopy (Hitachi 7000-FA).

### Expression and purification of the recombinant VP5 and VP7 proteins in *E. coli*

To produce purified recombinant VP5 and VP7 on a large scale, the amplified genes of interest were cloned into the pRSET-A vector (named as pR/GCRV-VP5 and pR/GCRV-VP7) [21,22]. The constructed recombinants were transformed into BL21(DE3) pLysS cells, which were propagated in a large scale culture. For time course expression, the cells were induced with 1 mM IPTG (Isopropyl β-D-1-thiogalactopyranoside) at 37°C. All the cultured bacteria were collected and lysed and the cell lysate extracts were resuspended in phosphate-buffered saline (PBS, 137 mM NaCl, 2.7 mM KCl, 8.1 mM Na_2_HPO_4_, 1.5 mM KH_2_PO_4_; pH 7.5). The suspensions were mixed with 2 × SDS sample buffer for further characterization by sodium dodecylsulfate-polyacrylamide gel electrophoresis (SDS-PAGE) as described elsewhere [16]. Because of the presence of a His-tag region in the N-terminus of the recombinant fusion protein, affinity purification of the protein could be performed. For the purification, the ProBond™ Resin Purification System (Invitrogen, USA) was used according to the manufacturer's instructions.

### Preparation of antisera and ELISA

The purified recombinant proteins VP5 and VP7 and the gradient-purified GCRV sample were used as antigens to raise antibodies in the New Zealand white rabbit. VP5Ab, VP7Ab and GCRV antibody were obtained by immunization of rabbits with purified native GCRV, His-VP5 and His-VP7 fusion proteins respectively. The titers of the antibodies were determined by using ELISA (Enzyme-linked immunosorbent assay) as described elsewhere [22]. Each antiserum dilution was assayed in duplicate with three repeats.

### SDS-PAGE and Western blotting analysis

The samples used for protein analysis were subjected to 12% sodium dodecylsulfate-polyacrylamide gel electrophoresis (SDS-PAGE) as described above. Proteins were visualized by using 0.25% Coomassie brilliant blue R-250 (Sigma). Western blotting analysis was used to detect the specificities of the expressed proteins. We used either His-tag (mouse) or GCRV, VP5, VP7 protein antibodies (rabbit) against the appropriate epitope as the primary antibody. Alkaline phosphatase-coupled rabbit anti-mouse and goat anti-rabbit IgG were used as the secondary antibodies. All the results were observed by developing with AP substrate solution (NBT/BCIP).

### Infection and determination of virus titer

GCRV was inoculated in CIK cells with a virus stock at 10 MOI (multiplicity of infection). The titer of GCRV was determined by using CPE-based TCID_50 _(50% tissue culture infective dose) or plaque assays as described elsewhere [24]. Virus stock was serially diluted 1:10 in 1 x PBS, and the diluted virus stock was inoculated in triplicate onto confluent monolayers of CIK cells. Infected cells were incubated at 28°C in MEM-2 (MEM with 2% FBS, named MEM-2) for 3 days. Virus infectivity was measured by calculating the TCID_50_, and the endpoints were calculated by the methods of Reed & Muench [25].

### Neutralization assay of prepared antibodies

Infectivity of antibody-treated virus was determined by both plaque and CPE-based TCID_50 _assays. Briefly, native GCRV antibody, VP5Ab and VP7Ab were heat inactivated at 56°C for 30 min and diluted with Eagle's MEM medium with doubling dilutions. Nine serial twofold dilutions (1:8 - 1:2048) were used for the neutralization test. Cells infected with 100 TCID_50 _GCRV virions that were titered as described above, and mock-infected cells served as positive and negative controls, respectively. For the neutralization assay, 100 μl antiserum and 100 μl virus (containing 100 TCID_50 _virions) were added to each well of 24-well microtiter plates and mixed gently, followed by incubating the mixture at 37°C for 60 min, and the next neutralization test was performed as described elsewhere [16,24]. The endpoints of neutralization antibody titers were determined by the Reed-Muench method [25]. For plaque assays, the specific reduction in virus infectivity was calculated as the percent of plaque reduction. TCID_50 _assays used for determining virus titers in this study were the averages obtained from triple determinations for each sample with three or four repeats.

## Results

### Analysis of protein components of GCRV particles

**Figure 1:**
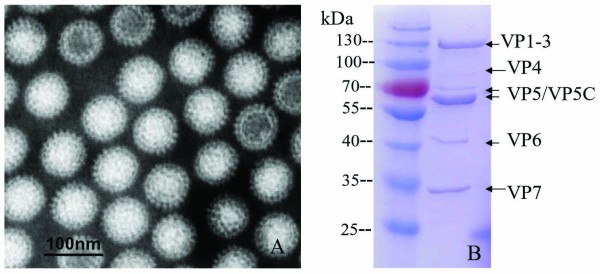
**Purified GCRV particles and their protein components**. A: Purified GCRV particles observed by TEM; B: SDS-PAGE analysis of GCRV structural proteins.

To identify purified virion preparations, the GCRV samples obtained by cesium chloride (CsCl) density gradient centrifugation from infected cell culture supernatant were observed using transmission electron microscopy (TEM). Purified virions had a clear double capsid shell morphology and structure organization with a size of approximately 80 nm in diameter (Figure 1A). To further verify the components of purified GCRV, the virus sample was analyzed by SDS-PAGE (Figure 1B). The purified virus sample appeared to contain seven capsid proteins VP1-VP7, indicating that the purified native GCRV preparation can be used as an antigen to immunize animals.

### Purification and identification of expressed recombinant proteins

**Figure 2:**
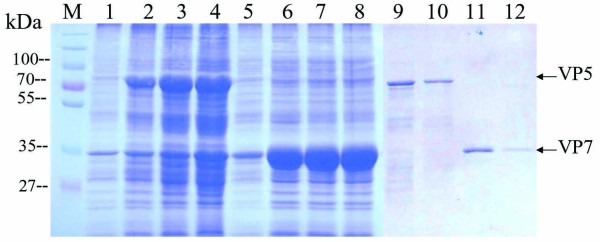
**Expression and purification of recombinant VP5 and VP7**. M, Standard protein marker; Lane 1 and Lane 5, empty vector control; Lanes 2-4, VP5 cell lysate extracts induced by IPTG for 1, 3 and 5 h, respectively; Lanes 6-8, VP7 cell lysate extracts induced by IPTG for 1, 3 and 5 h, respectively; Lanes 9-10, purified VP5; Lanes 11-12, purified VP7. All of the total cell extracts and purified proteins were electrophoresed on a 12% SDS-polyacrylamide gel followed by staining with 0.25% Coomassie brilliant blue R-250.

Previously, we have successfully expressed VP5 and VP7 protein in *E. coli *[17,18]. To make a large-scale preparation for further immunogen analyses, the highly expressed recombinant His-tag fusion proteins of VP5 and VP7 were purified using the ProBond™ resin purification kit. As shown in Figure 2, the purified VP5 (about 70 kDa) and VP7 proteins (about 35 kDa) were the products of interest. Western blotting analysis showed that expressed His-tag fusion proteins VP5 and VP7 could bind to the anti-His-tag antibody(mouse), demonstrating that the *in vitro*-expressed recombinant products were the His-tag fusion proteins of interest (data not shown).

### Titration of prepared antisera and specific analysis

**Figure 3:**
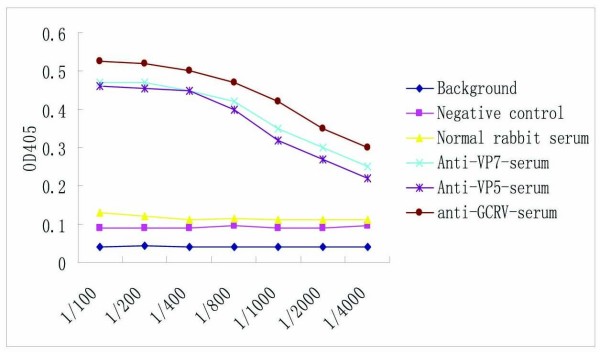
**Titration of the polyclonal VP5Ab, VP7Ab and GCRV antibody by ELISA**. All the tested samples are marked with different colors.

Purified recombinant VP5 and VP7 proteins and native GCRV particles were immunized into the New Zealand white rabbit. After twice-enhanced immunization, the primary titer of the antiserum was tested. Further titers of VP5Ab, VP7Ab and GCRV antibody were determined with antigen-coated GCRV particles using ELISA. It was found that the minimum *OD*_405 _values of polyclonal anti- GCRV, VP5 and VP7 sera at a dilution of 1:1000 were above 0.42, 0.32 and 0.35, respectively, which was about 3 times higher than the value for normal rabbit serum (Figure 3), indicating that the *in vitro*-expressed VP5 and VP7 had good immunogenicity.

**Figure 4:**
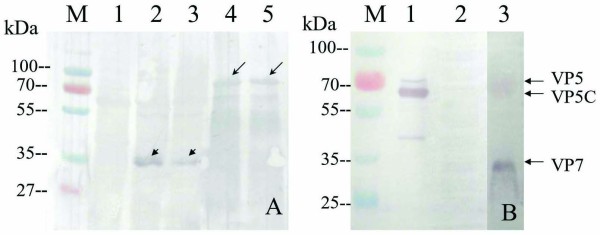
**Western blotting analysis of expressed proteins VP5, VP7 and GCRV**. **A:** Western blotting analysis of expressed VP5 and VP7 cell lysate extracts in *E. coli *with native GCRV antibody. M, Standard protein marker; Lane 1, empty vector cell lysate extracts induced with IPTG for 3 h as negative control; Lanes 2-3, VP7 cell lysate extracts induced by IPTG for 3 h; Lanes 4-5, VP5 cell lysate extracts induced with IPTG for 3 h. The long arrowhead indicates VP5 and short arrowhead indicates VP7. **B**: Western blotting of purified GCRV with VP5Ab and VP7Ab; Lane 1, purified native GCRV particles with VP5Ab; Lane 3, purified native GCRV particles with VP7Ab; Lane 2, un-infected CIK cell as negative control.

To further access whether the prepared VP5 and VP7 polyclonal antisera could be recognized by native GCRV antigen, western blotting analyses were performed. It appeared that purified VP5 and VP7 recombinant proteins could bind to the GCRV antibody; no visible band could be detected from the expression of empty vector, demonstrating that the expressed VP5 and VP7 proteins were specific to GCRV antibody, as shown in Figure 4A. Furthermore, GCRV particles could be immunologically recognized by VP5Ab and VP7Ab, whereas un-infected CIK cell lysate had no cross-immunological reaction with VP5Ab and VP7Ab, suggesting that the VP5Ab and VP7Ab are specific to GCRV.

### Neutralization assay of tested antibodies

As indicated above, GCRV antibody, VP5Ab and VP7Ab all had comparatively high titers according to ELISA detection. To evaluate the neutralization ability of the prepared antibodies to GCRV infection, neutralization assays were performed with GCRV antibody, VP5Ab, VP7Ab individually and VP5Ab and VP7Ab in combination, respectively. The neutralization titers of four tested sera varied in the following order: native GCRV > VP5+VP7 > VP7 > VP5 (as shown in table 1). The 50% neutralization titer of the single VP7Ab was 1/210, which was 3 times higher than that of VP5Ab. This suggests that the VP7 protein might be the dominating epitope, and VP7 expressed in *E. coli *has good immunogenicity. Moreover, the combination of VP5Ab and VP7Ab had a 50% neutralization titer of 1/350, hinting that VP5Ab may have a compensatory effect to neutralize intact GCRV particle or ISVP infectivity.

**Table 1:** Comparison of the neutralizing titers of tested antibodies

Antibody specimen	Serum dilution	Percent of CPE*/plaque	50% Neutralization titer
Native GCRV antibody	1/256	11%	1/460(10^-2.66^)
	1/512	56%	
	1/1024	100%	
VP5Ab	1/64	45%	1/70(10^-1.85^)
	1/128	73%	
	1/256	100%	
VP7Ab	1/128	33%	1/210(10^-2.325^)
	1/256	56%	
	1/512	100%	
VP5Ab+VP7Ab	1/256	42%	1/350(10^-2.55^)
	1/512	67%	
	1/1024	100%	

*CPE, cytopathic effect.

## Discussion

Non-enveloped virus attachment and entry into host cells are multiple steps that influence cellular tropism and can involve sequential recognition of multi-receptors. Studies on the GCRV genome and 3D structure have revealed that the VP5 and VP7 proteins comprise the outer capsid shell of the virus, which resemble the μ1 and σ3 proteins in MRV, respectively, in both protein homology and particle location. Similar to MRV μ1 and σ3, the outer-capsid proteins VP5 and VP7 play critical roles in virus entry [13,15,26]. In MRV, the first step in entry is binding to cell surface receptors, which is mediated by the σ1 trimer located at each fivefold axis in the virions [27]. Due to the lack of the σ1 protein in GCRV, the outermost protein VP7 may function to bind cell receptors to initiate infection. Following binding, the major outer-capsid proteins VP7 and VP5 are proteolytically cleaved to yield metastable ISVP. This leads to VP5 structural rearrangement through conformational change in order to penetrate cell membrane.

We found that the neutralizing activity of VP7 antiserum was 3 times higher than that of VP5 antiserum, which was also confirmed by single chain fragment of variable (scFv) antibodies against Grass Carp IgM (Chen *et al*., unpublished data), suggesting that VP7 might be a major antigen determinant. Given this result, it is proposed that VP7 may act as a cell attachment protein to bind to the cell receptor, because VP7 is located at the outermost layer of the particle. The effective combination between antigen and antibody may block out the cell attachment site and prevent virus infection. It appeared that the neutralization titers induced by VP5 and VP7 (1:70 and 1:210, respectively) were lower than that of native intact virus (1:460). This may be due to a variation in VP5 and VP7 protein conformation or folding in *E. coli *expression from that of the natural VP5 and VP7. The neutralization titer in combination with VP5Ab and VP7Ab was higher than that of a single antibody, suggesting that GCRV infection is not dependent upon a single viral protein or cell factor. In this way, VP7 antiserum may target neutralizing intact particles, while VP5 antibody is preponderant to neutralize ISVP. The fact that the neutralization titer of VP5Ab and VP7Ab combined was higher than that of single antibody suggests that there is one more receptor-binding site on GCRV surface proteins. Furthermore, the result is also consistent with a previous report that revealed that removing the outermost capsid protein (VP7) of an aquareovirus was associated with increased infectivity [28], indicating that the VP5 and VP7 proteins of GCRV are possibly involved in initiating viral infection. Notably, sequence alignment and structural comparison with λ2 in MRV suggest that the GCRV VP1 turret structure can be divided into a GTase(guanylyltransferase)domain, two methylase domains, and an immunoglobulin (Ig)-like flap domain. The most significant difference between MRV λ2 and GCRV VP1 was observed in the Ig-like flap domain. In this regard, it is postulated that the VP1 flap domains might also be involved in conferring host specificity during virus entry into cells.

In this study, we prepared GCRV, VP5 and VP7 polyclonal antibodies generated from purified native GCRV, recombinant VP5 and VP7. Our results indicated that the prepared antibodies were specific and immunologically related to the GCRV epitope, and cross-reaction of the antibodies to GCRV particles could be detected by western blotting analysis. In addition, both VP5Ab and VP7Ab were capable of neutralizing viral infectivity, evaluated using CPE-based TCID_50 _and plaque assays. Moreover, the neutralizing activity of VP7 antiserum was 3 times higher than that of VP5Ab, suggesting that VP7 might be the dominating epitope. Furthermore, the combination of VP5Ab and VP7Ab presented an enhanced neutralizing capacity to GCRV. The data can be used to further define the surface epitope domains of GCRV and may have significant applications for vaccinating against GCRV infection.

## Conclusions

The results presented in this study showed that the antiserum of VP7 protein had a higher neutralization ability than that of VP5 antiserum, while the combination of VP5Ab and VP7Ab had a stronger capacity to neutralize GCRV than either antibody singly. This study not only lays a foundation for further vaccine design, but also provides a framework for further investigation of the neutralization mechanisms of aquareoviruses.

## Competing interests

The authors declare that they have no competing interests.

## Authors' contributions

QF designed the experiments. QF & LS analyzed the data and wrote the paper. LS, XYS, and QF carried out all the experiments. All authors read and approved the final manuscript.
